# Short-Term Selection to Diflubenzuron and *Bacillus thuringiensis* Var. *Israelensis* Differentially Affects the Winter Survival of *Culex pipiens* f. *Pipiens* and *Culex pipiens* f. *Molestus* (Diptera: Culicidae)

**DOI:** 10.3390/insects12060527

**Published:** 2021-06-06

**Authors:** Charalampos S. Ioannou, Christos Hadjichristodoulou, Maria A. Kyritsi, Nikos T. Papadopoulos

**Affiliations:** 1 Laboratory of Hygiene & Epidemiology, Faculty of Medicine, School of Health Science, University of Thessaly, 41222 Larissa, Greece; ioannoubabis@yahoo.com (C.S.I.); xhatzi@med.uth.gr (C.H.); mkiritsi@med.uth.gr (M.A.K.); 2 Laboratory of Entomology & Agricultural Zoology, Department of Agriculture Crop Production and Rural Environment, School of Agricultural Sciences, University of Thessaly, 38446 Volos, Greece

**Keywords:** common house mosquito, diapause, insecticides efficacy, mosquito control

## Abstract

**Simple Summary:**

In Europe, *Culex pipiens* (Diptera: Culicidae) mosquito, the prime vector of West Nile virus, consists of two forms, named *pipiens* and *molestus*, that exhibit substantial differences in their biology, including overwintering behavior. Diflubenzuron (DFB) and *Bacillus thuringiensis* var. *israelensis* (*Bti*) are among the most widely used larvicides which pose major concerns for resistance development. In temperate areas, winter represents a very challenging period for the survival of many insects, including mosquitoes, and therefore potential fitness costs associated with insecticide selection may reduce their overwintering success. In this context, we investigated how short-term selection of *Cx. pipiens* f. *pipiens* and *molestus* forms to DFB and *Bti* affect their overwintering success. Our findings revealed that selection to both larvicides induced a high fitness cost in terms of reduced winter survival of *Cx*. *pipiens* f. *molestus* but not of *pipiens* form, suggesting potential differences in the persistence of the selected individuals in the wild from year to year.

**Abstract:**

The *Culex pipiens* (Diptera: Culicidae) mosquito is of high medical importance as it is considered the prime vector of West Nile virus. In Europe, this species consists of two forms, named *pipiens* and *molestus*, that exhibit substantial differences in their overwintering biology. Diflubenzuron (DFB) and *Bacillus thuringiensis* var. *israelensis* (*Bti*) are two of the most used larvicides in mosquito control, including that of *Culex pipiens*. The high dependency on these two larvicides poses major concerns for resistance development. The evolution and stability of resistance to insecticides has been associated with fitness costs that may be manifested under stressful conditions, such as the winter period. This study investigated how short-term selection of *pipiens* and *molestus* forms to both larvicides affect their overwintering success. Larvae from each form were subjected to the same selective pressure (80% mortality) for three successive generations with DFB and *Bti*. At the end of this process, the winter survival between the selected populations and the controls (colonies without selection) was determined for each form. Selection to both larvicides significantly reduced the winter survival rates of *molestus* but not of *pipiens* form, indicating potential differences in the persistence of the selected individuals from year to year between the two forms.

## 1. Introduction

The common house mosquito, *Culex pipiens* (L.), is a widespread insect pest of medical and veterinary importance as it is considered an effective vector of several human and animal diseases, including filarial nematodes and arboviruses such as West Nile virus (WNV), Sindbis virus, Rift Valley fever and Japanese encephalitis virus [1,2]. *Culex pipiens* includes two distinct forms (usually referred to as biotypes), *pipiens* and *molestus*, which are morphologically identical but differ in several behavioral and physiological aspects [3,4]. In particular, the *molestus* form prefers to colonize underground breeding sites, while *pipiens* is commonly found in above-ground habitats. Moreover, *Cx*. *pipiens* f. *molestus* is stenogamous (copulation can occur in confined spaces), autogenous (ability to develop a first batch of eggs without a blood meal) and mammophilic (prefers to feed on mammals, including humans). On the other hand, *Cx*. *pipiens* f. *pipiens* is eurygamous (copulation occurs outdoors in swarms), anautogenous (blood feeding is necessary for eggs’ development) and rather ornithophilic (prefers to feed on birds) [3]. Another major difference between the two forms is in their winter biology at temperate regions. Contrary to the *molestus* form, which remains active and reproduces during winter, the *pipiens* form undergoes diapause as inseminated females with arrested ovariole development and elevated fat body reserves that serve as an energy source [3,5,6]. Short day length and relative low temperatures perceived in larval and pupal stages during autumn are responsible for triggering the physiological changes underlying diapause induction [7,8]. 

Although nowadays several non-chemical methods for mosquito control are under development and evaluation, such as the Sterile Insect Technique (SIT), the Release of Insects carrying a Dominant Lethal (RIDL) and the release of *Wolbachia*-infected mosquitoes [9], insecticide applications still remain the principal tool for tackling mosquito-related problems. Among the insecticides that are employed, larvicides are considered as the most important means for the prevention of mosquito-borne diseases, as they target immature stages (larvae and pupae) and thus prevent females’ emergence, which are responsible for pathogens’ transmission. Despite the high importance of this approach, under the current European Union biocide legislation and the prohibition of organophosphates (OPs) such as temephos, larval control relies almost exclusively on two main categories of biocides, the insect growth regulators (IGRs) and the microbial control agents [10]. Diflubenzuron (DFB) and *Bacillus thuringiensis* var. *israelensis* (*Bti*) are the most widely used larvicides in each category, as they combine some very desirable features, such as the high efficacy against mosquito larvae and the very low toxicity to vertebrates. Diflubenzuron inhibits the chitin biosynthesis process, causing abnormal molting during the immature development, preventing adult emergence [11]. On the other hand, during sporulation, *Bti* produces four major (Cry11Aa, Cry 4Ba, Cry 4Aa and Cyt1Aa) and at least two minor (Cry10Aa and Cyt2Ba) toxins [12]. The Cry toxins bind to specific midgut membrane receptors, disrupting its integrity, while the Cyt toxins appear to act synergistically with the Cry toxins, functioning as surrogate receptors that improve their capacity to bind on the available target sites (receptors) [13,14]. The synergistic interactions between the Cyt and the Cry toxins are considered the key factors for the low potential of resistance development in mosquitoes following extensive selection with *Bti* [12,15].

The high dependency on both DFB and *Bti* for the suppression of mosquito populations, including *Cx*. *pipiens*, poses major concerns for resistance development, which may jeopardize the control efforts and increase the risk of disease transmission, such as WNV. Indeed, striking DFB resistance levels associated with specific mutations have already been detected in *Cx*. *pipiens* natural populations from Italy and Turkey in areas with intense use of this insecticide [16,17,18,19]. On the other hand, there is only a single record of high (33-fold) *Bti* resistance levels in *Cx*. *pipiens* wild populations, with a background of previous exposure to support for potential resistance development [20]. Interestingly, evaluation of field populations of *Cx*. *pipiens* without history of *Bti* exposure has shown variations of resistance ratios (RR) ranging from less than 3- to 10-fold [21]. Therefore, this inherent variability in *Cx*. *pipiens* populations’ susceptibility to *Bti* may be of high importance since it is possible to affect their response to selection pressure. Despite the prominence of the above findings regarding both DFB and *Bti* resistance in *Cx*. *pipiens* populations, no efforts have been made to separate potential differences between the forms *pipiens* and *molestus* given their divergent biology.

Insecticide resistance emergence and evolution is a dynamic process that depends, among others, on the genetic background and the biology of mosquito species, the intensity of biocide selection pressure and the resistance mechanisms that are involved [22]. However, resistance development is often associated with significant reductions in fitness parameters of the resistant individuals as a result of increased metabolic costs of physiological/biochemical resistance mechanisms [23]. Moreover, adverse abiotic conditions can also affect the impact of insecticide resistance on key life history traits. Winter in temperate areas represents a very challenging and stressful period for the survival of many insects, including mosquitoes, and therefore potential fitness costs associated with insecticide resistance may reduce their overwintering success. Indeed, the frequency of two genetic loci in *Cx*. *pipiens* f. *pipiens* females associated with OPs resistance decreased over winter, indicating reduced survival for resistant individuals [24]. This is of great importance since it can determine the persistence of resistant mosquitoes from year to year, affecting both the evolution and stability of acquired resistance in the wild. However, it remains completely unknown if a similar phenomenon holds in the case of DFB and *Bti* given their different mode of action relative to OPs.

Considering the importance of *Cx*. *pipiens* as an effective vector of several diseases, the fundamental differences in biology between its two forms and the high dependency on DFB and *Bti* to control its populations, the aim of the present study was to explore how short-term selection of *pipiens* and *molestus* forms to both larvicides affect their susceptibility, and most importantly, their overwintering success. For this purpose, we established colonies of both *Cx*. *pipiens* f. *pipiens* and *Cx*. *pipiens* f. *molestus* originated from the same area and subjected them to the same selective pressure (80% population mortality) for three successive generations with DFB and *Bti*. This process intended to simulate a more or less representative selective pressure that is imposed during a single control season. Despite the intensive larvicide applications that may occur in the field, in most cases, this does not ensure a strong, uniform selective pressure against *Cx*. *pipiens* populations. The divergent natural and anthropogenic breeding sites that the species utilizes, including the below-ground ones (*molestus* form), inevitably suggests that certain proportions of *Cx*. *pipiens* populations would always avoid exposure. In this concept, the adopted selection protocol resembles a more realistic approach on the actual selective pressure experienced by natural populations of *Cx*. *pipiens* at the end of a control season and the beginning of the winter period. After the completion of the selection process, the susceptibility levels and the overwintering survival of the selected populations relative to controls (colonies that received no selection) were determined.

## 2. Materials and Methods

### 2.1. Mosquito Colonies and Rearing Methods

All mosquito colonies were established during early September to late October of 2017 from eggs that were collected at the vicinity of Volos and Larissa city, Thessaly province, Greece. To collect the egg rafts, we established a grid of 20 (10 per city) green, plastic containers provided with 15 L of tap water. Containers were placed at a distance of 300–500 m from each other in well-protected spots from direct sunlight and strong winds. Half of the containers were placed in backyards and the other half in close proximity (10–20 m) to hen coops and pigeon houses. Earlier surveys in the study area revealed *Cx*. *pipiens* as the most abundant mosquito species, followed by *Aedes caspius* [25]. Since the 2010 WNV outbreak in Greece, human cases regularly occur in the area, with a peak of 24 incidents in Larissa in 2019 [26]. Mosquito control has been performed routinely since 2010 by private enterprises and involves mainly the use of DFB in urban and suburban breeding sites, while *Bti* is applied in protected wetlands such as rivers, streams and lakes [18,27]. A total of 74 and 59 egg rafts were recovered for *Cx*. *pipiens* form *pipiens* and *molestus*, respectively. Separation of *Cx*. *pipiens* forms was confirmed by matrix-assisted laser desorption/ionization time-of-flight mass spectrometry (MALDI-TOF MS) protein profiling [28]. Colonization took place within the insectary facilities of the laboratory of Entomology and Agricultural Zoology at the University of Thessaly. The insectary walk-in chamber was maintained at 25 ± 1 °C, 65% ± 5% relative humidity and a photoperiod of L14:D10, with a simulated dusk and dawn for 45 min. Photophase initiation was set at 00:00 h and termination occurred at 14:00 h. Larvae were reared in 42 × 30 × 10 cm^3^ white plastic containers in 3 L of bottled table water (Table Water, Epirotic Bottling Industry S.A. Ioannina, Greece), fed a total amount of 2 g of ground cat food (Friskies Adult, Purina, Italy) and held at a density of approximately 1000 individuals per container. Adults were kept in 32 × 32 × 32 cm^3^ screened cages at a density of 400–500 individuals, and fed with 10% sugar solution that was renewed every week. Females of *Cx*. *pipiens* f. *pipiens* were fed on certified human blood derived from samples that were provided by a blood analysis laboratory. Blood temperature was set at 38 °C using two custom-made, cylindrical (7.5 cm in diameter and 10 cm in height) feeding apparatus operated with circulating water from a warm bath via 12 V DC mini water pumps. The apparatus was placed on the top of the holding cages for 1 h, and females had access to feed via a stretched Parafilm M (Bemis, Neenah, WI, USA) membrane. Depending on the experimental needs, colonies received two to three blood meals per month. In general, colonies of *Cx*. *pipiens* f. *molestus* were kept without access to blood. Only in a few cases were they provided with a blood meal after the deposition of their first autogenous egg raft in order to reinforce colonies’ population during the selection process (see below). Both mosquito populations were reared for 3 generations in order to establish a uniform genetic background before the initiation of the experiments.

### 2.2. Larval Bioassays

Standard World Health Organization (WHO) guidelines [29] were adopted to evaluate the susceptibility of each collected population against DFB and *Bti*. Larval susceptibility was evaluated against analytical standard DFB (Purity ≥ 99.8%, Pestanal^®^, Sigma-Aldrich, Taufkirchen, Germany) and formulated *Bti* (Vectobac^®^ 12AS, 11.61% *w/w Bti* serotype H-14, strain AM65-52, 1200 ITU/mg, Valent BioSciences Corporation, Libertyville, IL, USA). Stock solutions were prepared in 99.5% acetone for DFB and distilled water for *Bti* and stored at −22 °C until use for up to two weeks. For DFB, six concentrations ranging from 0.001 to 0.01 mg/L and for *Bti* five doses ranging from 0.02 to 0.04 mg/L were used, yielding larval control between 10% and 95%. Six replicates were performed per concentration and an equal number of controls, each involving twenty-five third and early fourth instar larvae for DFB and *Bti*, respectively. Bioassays were run three times on different days using new batches of larvae and larvicide solutions. Larval mortality for *Bti* and adult emergence inhibition for DFB were recorded according to WHO recommended exposure times [29]. For each mosquito population, dose mortality responses were used to calculate the adult emergence inhibition (IE_50_, IE_80_ and IE_90_) values for DFB and the lethal concentration (LC_50_, LC_80_ and LC_90_) values for *Bti* using Probit Analysis [30].

### 2.3. Larval Selection

Larvae from each population were exposed for three successive generations to fixed concentrations of DFB and *Bti* corresponding to IE_80_ and LC_80_, respectively. During the selection process, six to eight groups of ≈1000 larvae were placed into rearing containers (see above) provided with 3 L of table water and the fixed dose of each larvicide. Selection against DFB involved third instar larvae, while 1.2 g of cat food was added in the containers to allow development (pupation). Selection against *Bti* involved the exposure of early fourth instar larvae to fixed doses for 24 h without any access to food. Surviving larvae were placed at a maximum density of ≈1000 individuals into rearing containers with 3 L of clean table water and offered 1 g of food to complete development. Resulting pupae from both DFB and *Bti* selection processes were transferred daily into cages and reared following the standard procedures described above. Additionally, for each population, two larvae groups (≈1000 individuals each) were maintained under identical conditions, but in the absence of DFB and *Bti* exposure serving as controls. There was at no point exchange of individuals among treatments and controls during the above-mentioned procedures. After the completion of the final (third) selection process, new dose–response bioassays were performed for each population to establish the new susceptibility levels against both DFB and *Bti*. A range of five concentrations was tested for each larvicide (DFB: 0.002–0.015 mg/L; *Bti*: 0.03–0.08 mg/L), using the same procedures described previously. Moreover, the winter survival of the descendants of both selected and control populations was evaluated.

### 2.4. Winter Survival

Since *Cx*. *pipiens* f. *molestus* remains active and reproduces during the winter period, both immature and adult survival were assessed. For this purpose, 1000 first instar larvae 3–5 h after their eclosion from both selected populations and the control were equally apportioned into five white, plastic containers (30 × 20 × 10 cm^3^) (200 larvae/container) with 1.5 L of table water and 2 mg of cat food per larvae. Then, all containers were randomly placed side by side in a humid, unheated warehouse located at the outdoor facilities of the laboratory that simulated the winter breeding sites of the species. Natural daylight was provided through a 70 × 70 cm^2^ window in the east side of the warehouse. To prevent water evaporation, each container was shielded with a well-fitted lid bearing a 2.5 cm hole at the center covered with mesh. Larval exposure took place on 23 December 2018. Containers were inspected every five days until the appearance of the first fourth instar larvae and daily after that. The initiation of pupation took place on 5 February and was completed on 3 March. Resulting pupae from each population were transferred into transparent, plastic bowls filed with 200 mL of table water kept at the same conditions. A maximum number of 15 pupae was placed in each bowl, while a lid prevented emerging adults from escaping. First adult emergence was observed on 17 February and the last on 5 March. Upon adult appearance, pairs consisting of a male and a female from each population were placed into individual cages. Each cage comprised a 0.4 L capacity transparent plastic cup (height 12.5 cm, upper diameter 6.5 cm, base diameter 9.2 cm) fitted into a 9.2 cm diameter plastic Petri dish lid. On the side of each cup, an opening of 25 cm^2^ covered with nylon mesh was formed for ventilation. Each individual cage was placed upon a 9 cm diameter Petri dish provided with 5% sugar solution, while adults had access to feed via a small piece of wick made of sponge cloth (Wettex^®^ classic, Freudenberg, Weinheim, Sweden). Depending on the adult emergence rates, 30, 47 and 50 replicates (pairs) were established for DFB and *Bti* selected and the control population, respectively. Adults were kept at the same place where larval development took place (warehouse). Their survival was monitored daily from 18 February until the death of the last individual on 27 June, while the sugar solution in the individual cages was renewed every week. Prevailing temperature and RH conditions inside the warehouse during the experiments were recorded by an indoor data logger (HOBO UX100-011, Onset Computer Corporation, Bourne, MA, USA) set to receive 4 recordings per 24 h. For each selected population and the control, both larval and pupal developmental duration and survival were assessed as well as adult longevity.

Diapausing females of *Cx*. *pipiens* f. *pipiens* from both selected populations and the control were reared from first instar larvae, using the standard procedures described above, in an environmental chamber set at 20 °C, 8L:16D and 70% RH [6]. Adults were provided constant access to 10% sugar solution 10–14 days post-eclosion to allow female copulation and lipid accumulation reserves. To confirm diapause induction, 10 randomly selected females from each population were dissected and the primary follicle/germarium length ratio was used as a criterion of ovarian diapause [31]. After the lipid accumulation period, two hundred females from each population were equally apportioned into five 20 × 20 × 20 cm^3^ screened cages (40 females/cage), having access only to water, and transferred to the same warehouse described above to simulate winter conditions. Female exposure took place on 15 January 2019. Cages were inspected at two-week intervals and female survival for each population was recorded. Dead mosquitos were removed with an aspirator through a 2 cm hole in the cage door shielded with a cork. Female winter survival was terminated at the end of March (26/3), following temperature rise, by providing the remaining individuals with 10% sugar solution without removing them from their winter shelter. Two weeks later, on 8 April, survived females from each population (25–32 individuals, see results) were merged in a 20 × 20 × 20 cm^3^ screened cage and given the opportunity to receive a blood meal for an hour over three successive nights. All three cages (one per population) were transferred inside the walk-in chamber between 20:00 and 22:00 h, and females were allowed to feed through the apparatus described above. After each blood feeding trial, cages were transferred back to the warehouse. One week following the last blood meal, a white, plastic cylindrical (10 cm in diameter and 5 cm in height) bowl with 200 mL of table water and 0.05 g of cat food was placed in each cage to allow oviposition. Bowls remained inside cages for 7 days. During that period, cages were inspected daily, and deposited egg rafts were collected and pictured under a binocular stereoscope (ZEISS, SteREO, Discovery.V12, Oberkochen, Germany) equipped with a digital camera (ZEISS, AxioCam, ERc 5s) to facilitate egg counting. Then, each egg raft was transferred individually into a white bowl (same as those that described above) covered with mesh to assess larval hatch rates under the warehouse conditions. The females of each population remained in the warehouse under ambient conditions and their survival was monitored every two days until the death of the last individual on 28 July, while the sugar solution in each cage was renewed every week. Climatic data (temperature and RH) during the course of the above experiments were recorded as described previously. For post-overwintering females of each population, we estimated (a) the percentage of individuals that received a blood meal, (b) preoviposition period, (c) mean number of eggs per raft, (d) mean larval hatch rate per egg raft and (e) lifespan under the ambient conditions inside the warehouse.

### 2.5. Statistical Analysis

The effect of mosquito population (DFB, *Bti* selected and control) on larval and female (in each sampling date) winter survival rates of *Cx*. *pipiens* f. *molestus* and *Cx*. *pipiens* f. *pipiens* respectively, were assessed using one-way analysis of variance, after appropriate transformations for normality and homoscedasticity when necessary, followed by Tukey’s HSD post hoc test to separate means. The same analysis was also performed to assess the effect of mosquito population on the number of eggs per raft and the larval hatch rates per egg raft of the survived *Cx*. *pipiens* f. *pipiens* females. The proportions of pupae survived in each population of *Cx*. *pipiens* f. *molestus*, their sex ratio as well as the proportions of female of *Cx*. *pipiens* f. *pipiens* that received a blood meal were analyzed using the Chi-square test. The effects of mosquito population and sex on larval and pupal developmental duration as well as on the adult lifespan of *Cx*. *pipiens* f. *molestus* were assessed using the Cox proportional hazards model. This model is commonly applied to assess the effects of one or more predictors on time to event incidents, such as time to pupation, adult emergence/death, etc. Pairwise comparisons were conducted using the log rank (Mantel–Cox) test. The same analysis was also performed to assess the effect of mosquito population on both the preoviposition period and the lifespan of the survived *Cx*. *pipiens* f. *pipiens* females. Data analysis was performed using IBM SPSS 25 (IBM Corp., Armonk, NY, USA).

## 3. Results

Dose–response larval bioassay results are provided in Table 1 and Table 2 for DFB and *Bti*, respectively. The EI_50_ values for DFB were similar between the two forms of *Cx*. *pipiens* before the initiation of the selection trials, however the EI_90_ value of *Cx. pipiens* f. *pipiens* was twice as much as that of *molestus*, suggesting a lower inherent susceptibility. The selection process for three successive generations by applying fixed DFB doses corresponding to the EI_80_ for each population resulted in 3.7 and 3.1 resistance ratio values for EI_50_ and EI_90_ respectively, for *Cx*. *pipiens* f. *pipiens*, and 1.7 and 2.9 for the *Cx*. *pipiens* f. *molestus* (Table 1). The LC_50_ and LC_90_ values for *Bti* were almost identical between the two populations before selection. The selection processes against *Bti* had minor effects on the susceptibility levels of both *Cx*. *pipiens* forms (Table 2).

**Figure 1:**
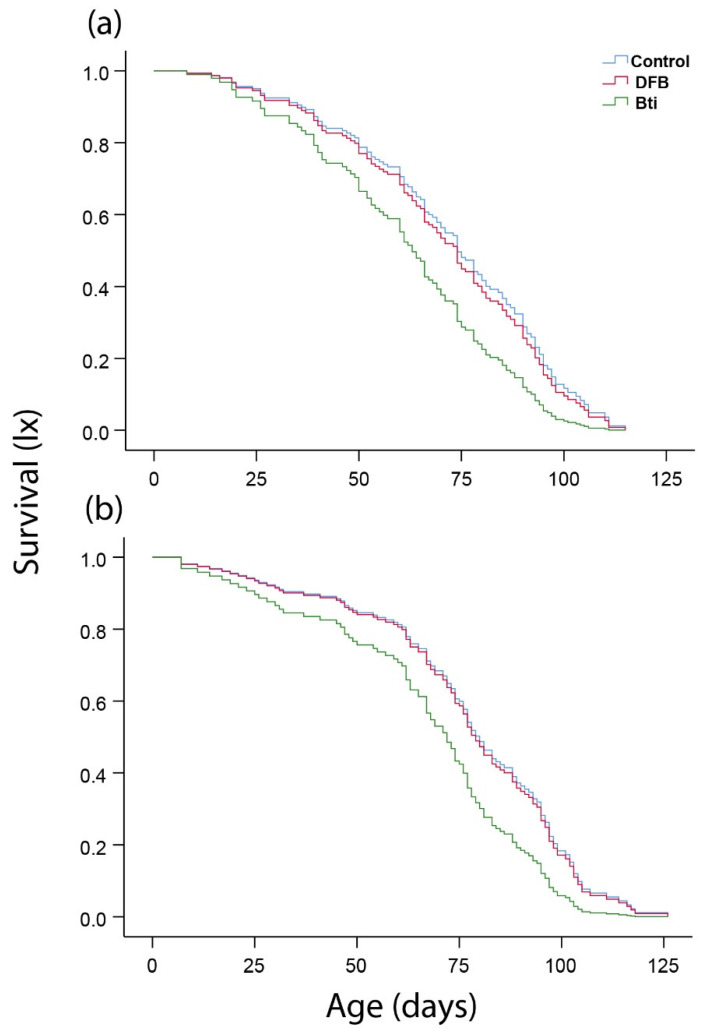
Male (**a**) and female (**b**) age-specific survival of *Culex pipiens* f. *molestus* populations that were either selected against diflubenzuron (DFB) and *Bti* for three successive generations or not (control). Adults were maintained in pairs (male and female) at the same place (warehouse) where they developed as larvae during winter, having access to 5% sugar solution. For the control, DFB and *Bti* selected populations, 49, 30 and 47 pairs were considered.

Ambient temperature and relative humidity conditions inside the warehouse from the beginning of the exposure of first instar larvae of *Cx*. *pipiens* f. *molestus* until the death of the last adult are depicted in Supplementary Figure S1. Temperatures ranged between 3.9 and 13.6 °C, with a mean value of 10.2 °C from larvae exposure (on 23 December) until the formation of the last pupae (on 3 March). Corresponding values regarding the first adult emergence until the death of the last individual ranged between 7.7 and 26.8 °C, with a mean value of 17.4 °C. Selection to both DFB and *Bti* significantly reduced larva-to-pupa survival relative to control, while significant differences were also observed between the two selected populations (Table 3). Cox regression analysis revealed mosquito population as a significant predictor of the larvae developmental duration considering the total number (viable and dead) of pupae formed. Larval developmental duration was significantly longer in both DFB and *Bti* selected populations relative to control, while significant differences were also observed between the two selected populations (Table 3). Pupal survival rates were higher in the control, however no significant differences were observed among the three populations (Table 3). Mosquito population was not a significant predictor of pupal developmental duration of *Cx*. *pipiens* f. *molestus* (Table 3). Sex ratio rates (females/males) were 52.3%, 58.3% and 44.3% for control, DBF and *Bti* selected populations respectively, though no significant differences were observed (Chi-square test, *X*^2^_2_ = 3.54, *p* = 0.170). Cox regression analysis revealed mosquito population (Wald test, *X*^2^_2_ = 13.29, *p* = 0.001) and sex (Wald test, *X*^2^_1_ = 4.52, *p* = 0.033) as significant predictors of adult lifespan. Both males and females of the *Bti* selected population exhibited significantly shorter longevity relative to control. Females of the DFB selected population also outlived those of *Bti* (Figure 1, Table 4).

**Figure 2:**
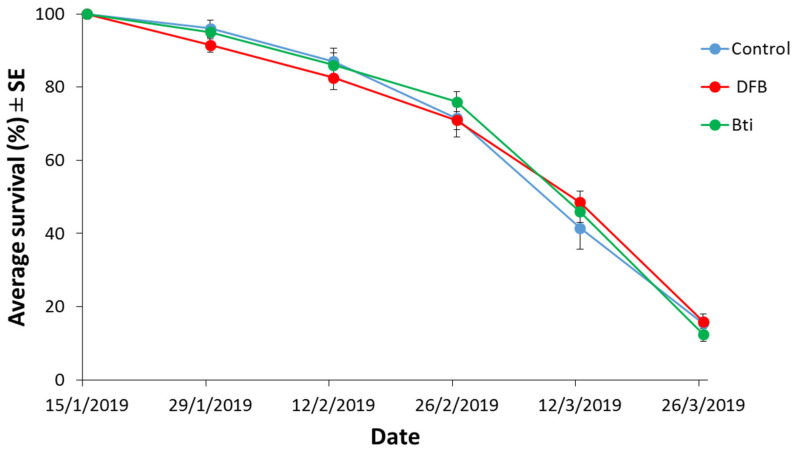
Winter survival rates of diapausing females of *Cx*. *pipiens* f. *pipiens* populations that were either selected against diflubenzuron (DFB) and *Bti* for three successive generations or not (control). For each population, five replicates were considered involving 40 females each.

Climatic conditions during the experiments of *Cx. pipiens* f. *pipiens* are shown in Supplementary Figure S1. Temperatures ranged between 7.7 and 16.5 °C, with a mean value of 11.9 °C from the beginning of female exposure until the termination of the overwintering period on 26 March. Corresponding values regarding the post-overwintering period until the death of the last female ranged between 13.5 and 27.1 °C, with a mean value of 21.2 °C. Analyses on each sampling date revealed no significant differences in female survival rates among the three populations (*F*_2, 12_ = 0.48 to 1.05, *p* = 0.379 to 0.631) (Figure 2). Additionally, no significant differences were observed in female reproductive parameters (Table 5). Finally, Cox regression analysis showed that mosquito population was not a significant predictor (Wald test, *X*^2^_2_ = 3.35, *p* = 0.187) of female post-overwintering survival. Average survival was approximately three months for all three populations, demonstrating that overwintered females of *Cx*. *pipiens* f. *pipiens* may experience extensive remaining lifespans (Table 6).

## 4. Discussion

Our study revealed that the implementation of equivalent selective pressure for three successive generations with both DFB and *Bti* against the two forms of *Cx*. *pipiens* induced differential susceptibility levels only in the case of DFB. The selected populations of *Cx*. *pipiens* f. *molestus* to both larvicides exhibited a high fitness cost in terms of reduced winter larval survival rates accompanied with increased larval developmental times. Moreover, the obtained adults of the *Bti* selected population appeared to suffer an additional cost in terms of shorter lifespan compared to the other two (control and DFB selected). On the other hand, the selection process had no apparent effect on *Cx*. *pipiens* f. *pipiens* female winter survival rates relative to control. Additionally, overwintered females showed similar reproductive parameters among populations. Interestingly, these females, irrespective of population origin, experienced considerable longevity during the post-winter period.

The EI_50_ values estimated in the current study for the control populations of both forms were found to be almost identical to those of the Benaki *Cx*. *pipiens* f. *molestus* laboratory reference strain [16], suggesting high susceptibility to DFB. Similarly, earlier studies conducted in different regions of Greece using the WHO diagnostic dose protocols demonstrated high susceptibility of most *Cx*. *pipiens* populations tested [27]. Moreover, recent surveys failed to detect any specific mutations in the chitin synthase gene of *Cx*. *pipiens* sampled from Greece that are associated with high levels of resistance against DFB. On the contrary, two and three different mutations at amino acid I1043 of the chitin synthase gene have been reported in neighboring countries Turkey and Italy respectively, with phenotypes exhibiting up to 128-fold resistance ratios (RR) relative to the Benaki reference strain [16,17,18,19]. The different selection pressure regimes imposed on these populations over the past years relative to the Greek ones have been proposed as the possible explanation. Interestingly, in our study, by imposing the same short-term selection pressure with DFB, we observed differential response of the susceptibility levels between the form *pipiens* and *molestus*, especially as EI_50_ RR values are concerned. This tendency was found to be even more pronounced after additional generations of continuous selection to DFB (F6, F9 and F12 generation) in the laboratory (Ioannou et al., in preparation). The reasons underlying this differential response to DFB selection of the two forms of *Cx*. *pipiens* remain largely unknown. It could be explained by their different biology. The tendency of the *molestus* form to reproduce below-ground may largely decrease both its exposure to insecticides as well as the gene flow rates among different populations. On the other hand, there are no such limitations for the above-ground free-living *pipiens* form. Therefore, it is anticipated that the *molestus* form populations may lack the genetic background for a rapid apparition and evolution of resistance. This argument is also supported by the fact that in the current study, the EI_90_ value of the control *Cx*. *pipiens* f. *pipiens* population (without selection) was twice as much as of the *molestus* form. Another possible explanation could be the fact that the DFB selected populations of each form may encounter differential levels of fitness costs in the wild, such as winter survival rates, as evidenced by the present study.

Short-term selection with *Bti* had a minor impact on the susceptibility levels of both biotypes. This is an expected outcome since there are no consistent recordings of mosquito resistance development to the full crystal. Despite some sporadic reports describing considerable resistance levels of wild mosquito populations against *Bti* [20,32,33,34], long-term studies under both laboratory and field conditions provide no support to these data [35,36,37,38,39,40,41]. The more or less unchanged susceptibility to *Bti* after long periods of applications in natural settings or intensive laboratory selection have been primarily attributed to the synergistic action between the Cry and the Cyt toxins, as previously mentioned. In contrast, selection with single, purified Cry toxins can rapidly lead to considerable resistance levels [35,42,43]. Another factor that seems to promote the lack of *Bti* resistance in mosquitoes is the fact that in the absence of selection pressure, within only a few generations (3–5), any acquired resistance disappears almost completely [36,38]. The high fitness costs following extensive selection to this microbial larvicide have been proposed as the most convincing explanation of this phenomenon.

Both DFB and *Bti* selection against *Cx*. *pipiens* f. *molestus* conferred a high fitness cost in terms of larvae winter survival, as mortality rates relative to control increased more than 50% and 30%, respectively. When we repeated the same experimental procedure after three additional generations of selection (F6) under optimum (laboratory) conditions, we found no apparent differences in larval survival rates among tested populations. This suggests that the observed costs are manifested only under stressful conditions. Indeed, the mean prevailing temperature during larval development reached the lowest developmental thresholds described for the species [44], shaping a very challenging environment for survival. In general, the fitness costs determined in optimal conditions are not always representative of that experienced in the wild. This is because stressful environments and/or limited resources might be more deleterious for resistant individuals. Similar to our findings, the prolongation of larval developmental times in OP-resistant *Cx*. *pipiens* populations was found to emerge only under stressful crowding conditions in natural breeding sites [45]. Therefore, both biotic and abiotic factors, as in our case, may shape the expression of fitness costs related to insecticide selection. The observed differences on the reduction of larval survival rates between DFB and *Bti* selected populations suggest deferential levels of fitness costs. A possible explanation may be found in the different mode of action of the two larvicides. An interesting finding that emerged from the current study is the fact that while short selection against *Bti* has minor effects on the susceptibility levels, it may confer a high fitness cost under stressful conditions. 

Reduction of larval winter survival rates following selection against DFB and *Bti* in *Cx*. *pipiens* f. *molestus* was also accompanied with a discrete increment of their developmental duration relative to control. Interestingly, the longest larvae developmental times were recorded in the *Bti* selected population. Moreover, the adults obtained exhibited significantly shorter lifespan compared to DFB selected and control populations. This is probably because selection to *Bti* has been associated with a modified microbiota of larvae, which may affect their proper functioning of the midgut and therefore the nutrients’ assimilation, developmental processes and ultimately adult performance, as it is well-documented that nutrition during the mosquito larval stage may shape important fitness elements of the emerging adults [46,47]. Similar to our results, *Bti* selection against *Ae*. *aegypti* also resulted in significant prolongation of larvae developmental times relative to control [38]. No effects were found regarding adult survival in the same study. However, it should be stressed that in this case, adults had access to only water and therefore no direct comparisons can be made with our results. A moderate reduction on both male and female longevity following selection with *Bti* was observed in *Cx*. *pipiens* f. *pipiens* compared to untreated control [36]. The optimal laboratory conditions in this study may account for not detecting significant differences.

Contrary to *Cx*. *pipiens* f. *molestus*, selection against DFB and *Bti* had no apparent effects on the winter survival of the *pipiens* form. The differential overwintering developmental stages (larva vs. adult) in these two forms may account for the observed outcome. Mosquito larvae require a minimum amount of nutrition to fully mature and pupate. Furthermore, larval developmental completion takes place within specific time limits, which are endogenously defined. This dynamic process may be more prone to selection costs relative to the adult stage, where full development has already been attained. In contrast to our findings, resistance of *Cx*. *pipiens* f. *pipiens* against OPs have been associated with reduced overwintering survival [24]. Among others, the differential exhaustion of fat reserves was proposed as the basic proximate cause. Indeed, a later study confirmed that the presence of resistance alleles against OPs in this species is negatively correlated with female lipid reserves [48]. The fact that female winter survival patterns in our study were almost identical between the control and the selected populations suggests no differences on the physiological process of fat reserves accumulation and/or exploitation. However, most importantly, it means that these females have equal probabilities of survival as the untreated ones, providing the base for the building of higher resistance levels in the next year. This argument seems to be supported by the fact that the resistance ratio against DFB of *Cx*. *pipiens* field populations in Italy, from 32.5-fold in 2015, reached 128.5-fold in 2016 [16], which backs the idea of the persistence of resistant mosquitoes in the wild from year to year. However, this is something that needs to be demonstrated for the specific populations, as in contrast to our case, they carry mutations of the chitin synthase gene associated with striking DFB resistance levels. Interestingly, further studies in the same country reveled a high focal distribution of DFB-resistant *Cx*. *pipiens* mosquitoes, which was attributed to the differential selection pressure imposed by both agricultural and mosquito control applications with DFB in the tested areas [17]. Since no separation between the two forms of *Cx*. *pipiens* took place in this study, the following explanation is possible. The high focal distribution of DFB-resistant mosquitoes may only reflect differences in the mosquito populations’ composition regarding the two forms, with the predominance of the *pipiens* form to account for the observed outcome. For instance, analysis of population structure from different areas of Greece, a country with identical climate, revealed extreme variations between the two forms on the composition of *Cx*. *pipiens* populations [49]. Nevertheless, as mentioned above, the validity of this hypothesis has to be confirmed for the specific populations. 

Winter survival of diapausing *Cx*. *pipiens* f. *pipiens* females may largely vary depending on the hibernacula conditions [3,50]. It has been observed that females may abandon their overwintering sites and actively search for new ones, a behavior described as an adaptive response associated with increased survival. It seems that the quality of each overwintering site, as experienced by females, is dependent on multiple parameters, such as the prevailing temperature and humidity levels, predator density, parasite frequency and human disturbance, and therefore this quality may change during winter [30]. Under our experimental design, mosquitoes were not able to select the most optimal environment for maximizing their survival as they were forced to overwinter in a given place (warehouse). Nevertheless, this does not diminish the reliability of our results since females from all populations experienced exactly the same conditions. Female survival rates observed in the current study are comparable with that found by Koenraadt et al. [50] considering similar overwintering sites, such as unheated house rooms. Interestingly, they found that non-diapausing females kept under the same conditions died within four days, suggesting that in the absence of nutritional resources, such females have a very limited ability to survive (but also see Rinehart et al. [51]). 

Reproductive parameters of overwintered females of *Cx*. *pipiens* f. *pipiens* were similar between the two selected populations and the control. Blood meal acceptance percentages ranged between 34.4% and 46.4%, suggesting that females had partially terminated their reproductive diapause by the time that trials took place. Contrary to other studies that used artificial conditions to terminate diapause in order to induce females to either respond to host stimuli [52] or receive a blood meal [50], we intentionally preferred to simulate as much as possible the natural conditions in an attempt to detect any potential variations in feeding activity. Although no significant differences were observed regarding both the preoviposition period and the average number of eggs per egg raft, the better performance of the control population with respect to these parameters may reflect an early form of fitness cost in the selected ones. The short-term selection to both larvicides may have acted against more pronounced differences, as indicated by other studies. For example, *Bti* selection against *Cx*. *pipiens* f. *pipiens* for 20 generations resulted in a 44.8% decrease of female fecundity [36], while Belinato and Valle [53], by applying the same experimental protocol as applied in this study, found that DFB selection against *Ae*. *aegypti* for 6 generations also conferred a significant reduction on this parameter. Finally, an interesting finding that emerged from the current study is the fact that females, irrespective of population origin (selected or not), experienced extensive post-overwintering longevity periods, which appeared to even exceed the total lifespans of *Cx*. *pipiens* f. *molestus* females (Table 3 and Table 5). This observation is in accordance with a previous study that documented that the physiological changes that take place during the pre-hibernation transition of these females confer a considerable increase in their longevity potential [54].

## 5. Conclusions

By imposing the same short-term selection with both DFB and *Bti* against *Cx*. *pipiens* forms *pipiens* and *molestus,* we found differences on the induced resistance ratios in the first case (DFB), which is probably associated with their divergent biology. Moreover, the selection process with both larvicides had a negative impact on the winter survival of the *molestus* form but not that of the *pipiens*. These findings provide insights into the early phase of resistance development between the two forms, but mostly on its prevalence in the wild from year to year. The incorporation of such information on a properly developed model is anticipated to contribute to the better understanding of the two *Cx*. *pipiens* forms’ performance against these two important larvicides over time.

## Figures and Tables

**Table 1:** Effective doses of diflubenzuron against the two forms of *Culex pipiens* after the selection process for three successive generations by applying fixed doses corresponding to EI_80_ of the control populations.

Population		N *	EI_50_ (95% CL) ^a^	RR_50_	EI_90_ (95% CL) ^a^	RR_90_	Slope	*X*^2^ (df)
*Cx. pipiens* f. *pipiens*	Control	3150	0.0025 (0.0014–0.0035)	-	0.0081 (0.0065–0.0097)	-	2.47	177.57 ^b^ (105)
	Selected	2700	0.0093 (0.0076–0.0106)	3.7	0.0252 (0.0218–0.0321)	3.1	2.96	70.98 (87)
*Cx. pipiens* f. *molestus*	Control	3150	0.0022 (0.0016–0.0026)	-	0.0040 (0.0037–0.0044)	-	4.49	201.99 ^b^ (105)
	Selected	2700	0.0037 (0.0028–0.0046)	1.7	0.0116 (0.0101–0.0132)	2.9	2.60	57.94 (87)

* Number of larvae tested. ^a^ EI values are expressed in milligrams per liter, and they are considered significantly different when 95% of confidence limits (CL) fail to overlap. ^b^ Since the goodness-of-fit test is significant (*p* < 0.05), a heterogeneity factor was used in the calculation of confidence limits (CL).

**Table 2:** Effective doses of *Bti* against the two forms of *Culex pipiens* after the selection process for three successive generations by applying fixed doses corresponding to LC_80_ of the control populations.

Population		N *	LC_50_ (95% CL) ^a^	RR_50_	LC_90_ (95% CL) ^a^	RR_90_	Slope	*X*^2^ (df)
*Cx. pipiens* f. *pipiens*	Control	2700	0.031 (0.029–0.033)	-	0.047 (0.044–0.051)	-	7.52	120.73 ^b^ (87)
	Selected	2700	0.042 (0.035–0.048)	1.3	0.069 (0.065–0.074)	1.5	6.12	47.99 (87)
*Cx. pipiens* f. *molestus*	Control	2700	0.032 (0.029–0.034)	-	0.047 (0.044–0.051)	-	7.62	126.68 ^b^ (87)
	Selected	2700	0.045 (0.034–0.059)	1.4	0.081 (0.065–0.089)	1.7	4.98	117.21 ^b^ (87)

* Number of larvae tested. ^a^ LC values are expressed in milligrams per liter, and they are considered significantly different when 95% of confidence limits (CL) fail to overlap. ^b^ Since the goodness-of-fit test is significant (*p* < 0.05), a heterogeneity factor was used in the calculation of confidence limits (CL).

**Table 3:** Fitness parameters of overwintering *Culex pipiens* f. *molestus* immature stages originated from populations that were either selected against diflubenzuron (DFB) and *Bti* for three successive generations or not (control). Numbers in parentheses indicate the individuals that were considered in each case. In each column, different letters indicate significant differences (*p* < 0.05).

Population	Larval Survival * ^a^ (% ± SE)	Larval Developmental Duration ^b^ (Days ± SE)	Pupal Survival ^c^ (%)	Pupal Developmental Duration ^b^ (Days ± SE)	
				Males	Females
Control	35.1 ± 1.77 ^a^	48.54 ± 0.17 ^c^ (351)	50.7 ^a^	11.75 ± 0.09 ^a^ (85)	11.89 ± 0.08 ^a^ (93)
F3 DFB	16.0 ± 1.58 ^c^	49.22 ± 0.20 ^b^ (160)	45.0 ^a^	11.50 ± 0.10 ^a^ (30)	11.64 ± 0.11 ^a^ (40)
F3 *Bti*	23.2 ± 2.31 ^b^	50.49 ± 0.32 ^a^ (232)	44.4 ^a^	11.57 ± 0.10 ^a^ (59)	11.87 ± 0.11 ^a^ (47)
*F*	25.26	-	-	-	-
*X* ^2^	-	30.26	2.10	3.38	1.42
df	2, 12	2	2	2	2
*p*	<0.001	<0.001	0.272	0.184	0.490

* For each population, five replicates were considered involving 200 first instar larvae each. ^a^ Tukey’s HSD test. ^b^ Wald test followed by log-rank test for pairwise comparisons. ^c^ Chi-square test.

**Table 4:** Longevity parameters of *Culex pipiens* f. *molestus* populations that were either selected against diflubenzuron (DFB) and *Bti* for three successive generations or not (control). Adults were maintained at the same place (warehouse) where they developed as larvae during winter, having access to 5% sugar solution. Within sex, different letters indicate significant differences (pairwise comparisons log-rank test, *p* < 0.05).

	Longevity Parameters in Days ± SE			
	Average	Quartiles		
		25	50	75
Males				
Control (*n* = 49)	72.88 ± 3.46 ^a^	92 ± 3.51	75 ± 6.29	60 ± 6.85
F3 DFB (*n* = 30)	69.13 ± 4.48 ^ab^	93 ± 6.95	66 ± 1.36	50 ± 9.68
F3 *Bti* (*n* = 47)	61.23 ± 3.69 ^b^	80 ± 4.83	68 ± 5.38	46 ± 11.45
Females				
Control (*n* = 49)	73.38 ± 4.37 ^a^	97 ± 1.72	77 ± 2.78	62 ± 13.39
F3 DFB (n = 30)	77.23 ± 4.50 ^a^	95 ± 6.17	76 ± 5.46	62 ± 3.22
F3 *Bti* (*n* = 47)	70.08 ± 3.33 ^b^	88 ± 3.59	77 ± 2.56	62 ± 7.44

**Table 5:** Reproductive parameters of post-overwintering *Cx. pipiens* f. *pipiens* females. Females originated from populations that were either selected against diflubenzuron (DFB) and *Bti* for three successive generations or not (control). In each column, different letters indicate significant differences (*p* < 0.05).

Population	N *	Blood Meal Acceptance ^a^ (%)	Preoviposition Period ^b^ (Days ± SE)	Eggs Per Raft ^c^ (±SE)	Larval Hatch Rate Per Egg Raft ^c^ (% ± SE)
Control	31	35.71 ^a^	10.00 ± 0.76 ^a^	61.10 ± 5.43 ^a^	89.10 ± 3.42 ^a^
F3 DFB	32	34.38 ^a^	11.72 ± 0.61 ^a^	51.54 ± 4.50 ^a^	92.58 ± 3.04 ^a^
F3 *Bti*	25	46.43 ^a^	11.53 ± 0.50 ^a^	51.46 ± 3.70 ^a^	90.68 ± 3.81 ^a^
*F*	-	-	-	1.43	0.22
*X* ^2^	-	1.06	2.64	-	-
df	-	2	2	2, 31	2, 31
*p*	-	0.587	0.266	0.254	0.798

* Number of females tested. ^a^ Chi-square test. ^b^ Wald test. ^c^ Tukey’s HSD test.

**Table 6:** Post-overwintering longevity parameters of *Cx*. *pipiens* f. *pipiens* populations that were either selected against diflubenzuron (DFB) and *Bti* for three successive generations or not (control). Females were maintained at the same place (warehouse) where they overwintered, having access to 10% sugar solution. Different letters indicate significant differences (pairwise comparisons log-rank test, *p* < 0.05).

	Longevity Parameters in Days ± SE			
Population	Average	Quartiles		
		25	50	75
Control (*n* = 31)	81.45 ± 6.02 ^a^	109 ± 6.59	89 ± 5.00	68 ± 15.02
F3 DFB (*n* = 32)	92.40 ± 5.84 ^a^	115 ± 1.23	105 ± 2.80	83 ± 22.04
F3 *Bti* (*n* = 25)	99.84 ± 3.19 ^a^	115 ± 1.62	97 ± 1.48	92 ± 2.91

## Data Availability

The data presented in this study are available upon request from the corresponding author.

## Supplementary Materials

The following are available online at https://www.mdpi.com/article/10.3390/insects12060527/s1, Figure S1: Ambient conditions inside the warehouse where winter and post-winter survival experiments of both *Culex pipiens* forms took place. White arrows indicate the initiation of larvae pupation and adult emergence of *Culex pipiens* f. *molestus*, respectively. Black arrows indicate the initiation and termination of the overwintering period of *Culex pipiens* f. *pipiens*.
