# Supplementary material for: Short-Term Selection to Diflubenzuron and Bacillus thuringiensis Var. Israelensis Differentially Affects the Winter Survival of Culex pipiens f. Pipiens and Culex pipiens f. Molestus (Diptera: Culicidae)
Source: Insects. 2021 Jun 6;12(6):527. doi: 10.3390/insects12060527 (PMC8228153; doi:10.3390/insects12060527)
Supplement: Supplementary file 1 [file insects-12-00527-s001.zip › insects-1203426-supplementary.pdf]

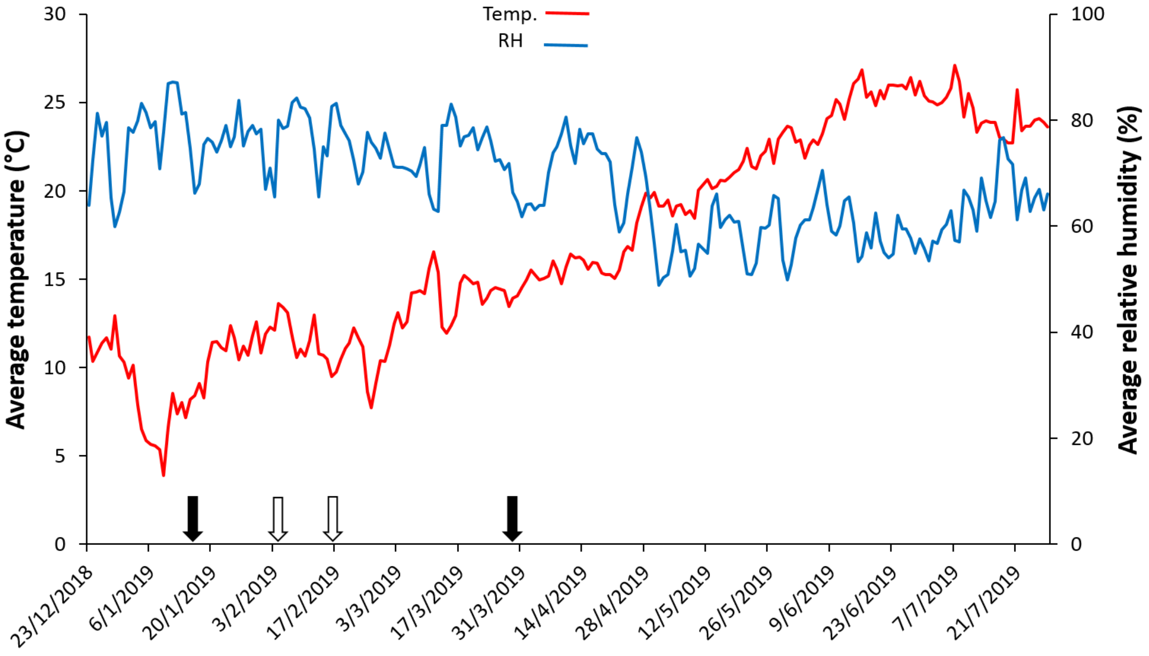


**Figure S1.** Ambient conditions inside the warehouse where winter and post-winter survival experiments of both *Culex pipiens* forms took place. White arrows indicate the initiation of larvae pupation and adult emergence of *Culex pipiens* f. *molestus,* respectively. Black arrows indicate the initiation and termination of the overwintering period of *Culex pipiens* f. *pipiens*.
